# Use of an E2SFCA Method to Measure and Analyse Spatial Accessibility to Medical Services for Elderly People in Wuhan, China

**DOI:** 10.3390/ijerph15071503

**Published:** 2018-07-17

**Authors:** Jing Luo, Guangping Chen, Chang Li, Bingyan Xia, Xuan Sun, Siyun Chen

**Affiliations:** 1 Key Laboratory for Geographical Process Analysis and Simulation, Central China Normal University, No.152 Luoyu Road, Wuhan 430079, China; luojing@mail.ccnu.edu.cn (J.L.); cgp@mails.ccnu.edu.cn (G.C.); xiabingyan@mails.ccnu.edu.cn (B.X.); sunxuan@mails.ccnu.edu.cn (X.S.); chensiyun@mail.ccnu.edu.cn (S.C.); 2 The College of Urban and Environmental Sciences, Central China Normal University, No.152 Luoyu Road, Wuhan 430079, China

**Keywords:** ageing population, medical services, spatial accessibility, enhanced two-step floating catchment area (E2SFCA), Wuhan city

## Abstract

Current studies on measuring the accessibility of medical services for the elderly (AMSE) have ignored the potential competition among supply and demand and the distance decay laws. Hence, an enhanced two-step floating catchment area (E2SFCA) method (i.e., the road network-based Gaussian 2SFCA method) is proposed to calculate AMSE scores after considering different types of roads, including urban rail transit, freeways, major roads, minor roads and rural roads. Based on the first National Geographic Conditions Monitoring (NGCM) data, this study took Wuhan, China, as a case study and assessed the variation of AMSE using two different threshold times (i.e., Platinum Ten and Golden Hour). Next, global (i.e., sensitivity and hot spot analysis) and local analyses (i.e., three regional area internal comparisons) of AMSE scores were conducted to accurately identify details in the variation of spatial accessibility. It was observed that the E2SFCA method could be easily applied to measure AMSE. The results showed that 48.63% of the elderly population in Wuhan had a higher or the highest level of medical accessibility in “Platinum Ten”, while 72.97% had a higher or the highest level in the “Golden Hour”, and hot spots of AMSE scores were located in central urban areas and presented an enclosure structure using both threshold travel times, which could provide guidance to governments or planners on issues of spatial planning and identifying elderly medical services shortage areas.

## 1. Introduction

The world population continues to age rapidly, as fertility rates have fallen to very low levels in many countries, and people tend to live longer [1]. In general, the trend towards older populations is largely irreversible [2]. The report *An Ageing World: 2015*, released by the U.S. Census Bureau in 2016, stated that 8.5% of the worldwide population was aged ≥65 years in 2015 and has reached 617 million, and this proportion is expected to almost double by 2050 [3]. An ageing population has significant, dynamic and complex healthcare needs [4]. The elderly, as a vulnerable population, need more accessible medical services. In 2002, the World Health Organization (WHO) released Active Ageing: a policy framework, which emphasized developing a continuum of accessible health services for the elderly [5]. Several organizations have suggested that priority action should be taken to ensure healthy aging and elderly people need more convenient medical services. Therefore, the spatial distributions of basic public medical service facilities need further optimization [6,7]. Additionally, considerable attention has been devoted to medical services for the elderly by geography. Several geographers have noted that planners should pay more attention to the elderly’s demands in the process of spatial planning of medical service facilities [8,9]. Because of the limited supply and spatial inequity of medical services in certain countries (especially developing countries) or regions, the contradiction between the supply and demand of medical services will always exist [10], but it can be alleviated through appropriate planning methods. Therefore, the work to measure and display the spatial accessibility of medical services for the elderly (AMSE) has become increasingly important.

Measuring spatial accessibility has received increasing attention in recent years. Also, the application of Geographic Information System (GIS) technology has recently been widely used to measure the transit access of public facilities (e.g., hospitals, schools, supermarkets, banks and so on) [11], and transport [12] based on single or multimodal transit network analyses [13]. Recently, a number of geographers have begun to focus on the measurement of AMSE. For instance, the nearest distance method is used to empirically measure and display the geographic accessibility of the aged population to hospital facilities [14]. Several researchers calculated car travel times to measure the accessibility to essential services (including hospitals) for the elderly according to an origin–destination (O–D) time cost matrix based on GIS road network analysis [15,16]. In other research, the Euclidean (straight line) distance was used to determine the medical service radius of the elderly in rural communities, and spatial allocation optimization of medical facilities was proposed [17]. Although these methods considered the factors of traffic time and distance from the medical services demand side to the supply side, they ignored the scale of both sides, and the travel behaviour of the elderly. Obviously, it would be desirable to use a more scientific approach for measuring AMSE.

The two-step floating catchment area (2SFCA) method, which is essentially a special case of the gravity model, is more intuitive to interpret and easier to calculate than the gravity model [18,19,20]. The 2SFCA method has been used in a number of recent studies that measured healthcare accessibility [21,22,23,24,25,26,27]. Despite many applications in several studies, the 2SFCA method has drawn sharp criticism because of its dichotomous approach that defines a doctor inside a catchment as accessible and one outside the catchment as inaccessible [28]. To overcome this shortcoming, a number of researchers have proposed an optimized method that added a Gaussian function to model the distance decay effect (i.e., a continuously gradual decay within a threshold distance or time), the results of which indicated that the Gaussian 2SFCA method performs better than the conventional 2SFCA [29,30]. In addition, with all forms of transport, elderly people are typically less likely to travel long distances [31]; specifically, they are more likely to obtain medical care near their residence. This phenomenon can be expressed mathematically as a Gaussian function in a distance (or time) decay mechanism. Recently, a Gaussian 2SFCA method has been employed in several practical studies [32,33,34,35,36,37]. However, notably little research has been conducted to measure the accessibility of ageing population-focused medical services using the Gaussian 2SFCA method, and in particular, very few studies of AMSE have considered the road network with different road types when calculating the travel times.

China is the world’s most populous country, currently having the largest number of elderly people, and the ageing trend of the population has accelerated in recent years. Official statistics indicate that mainland China now has 241 million elderly people, and the ageing proportion has reached 17.30% [38]. Wuhan is one of the largest ageing population cities in China and had already become an ageing society in 1993. The total population of elderly people has reached 1.79 million, and the ageing rate was 20.95% by the end of 2017. Medical services for the elderly have been becoming a critically important issue for the local government of Wuhan and society. Hence, Wuhan was taken as a case study in this paper. It is worth mentioning that the elderly’s medical services are mostly provided by public healthcare institutions (e.g., public general hospitals) in Wuhan. Public hospitals have a higher average value of total assets, more pieces of expensive medical equipment, more employees, and more physicians compared to private hospitals [39]. Therefore, public general hospitals in Wuhan were selected as the dominant medical services providers for elderly people. Overall, measures and analysis of AMSE in Wuhan, China, using the E2SFCA method could be helpful to the governments or planners monitoring the spatial inequity of the AMSE scores and could provide strategic references for further improvement of medical services for the elderly. Moreover, the application innovations and contributions of this paper are as follows:

1. The E2SFCA (i.e., the road network-based Gaussian 2SFCA) method is first proposed for measuring the AMSE. Based on the road network dataset, the travel time from urban–rural resident autonomous units (URAUs) to general hospitals for the elderly to access medical services was calculated using the O-D time cost matrix. Meanwhile, a Gaussian function was applied to improve the conventional 2SFCA for measuring the AMSE.
2. Ageing population-focused medical services accessibility measurement was first applied to Wuhan, China. Perhaps it can also be applied in other cities or even other countries in future studies.
3. Global and local analyses were conducted to comprehensively illustrate the spatial differences in AMSE scores. In terms of global analysis, sensitivity analysis uncovered differences in AMSE for two different threshold times. Hot spot analysis was used to assess whether and where high or low AMSE scores clustered spatially and to find hot spots, and in local analysis, the spatial differences in AMSE scores of three regional areas (i.e., central urban districts, development zones and new urban districts) were internally compared.

## 2. Study Area and Data

### 2.1. Study Area

**Figure 1:**
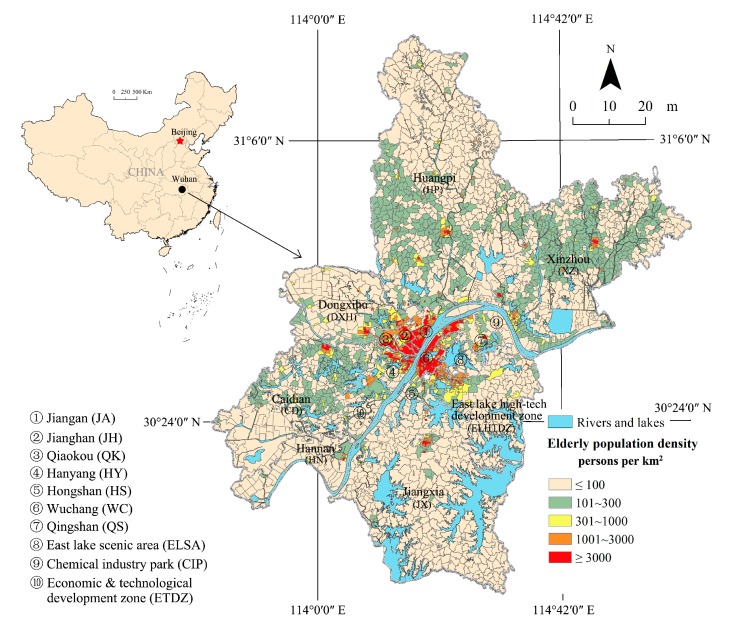
The elderly population density of Wuhan city in 2016.

Wuhan is the capital of Hubei province, which is located in central China and is a strategic pivot city for the region. Wuhan is one of many major cities in the Yangtze River delta and is also the core city of the National Central City and the Yangtze River Economic Belt. Because of its key role in domestic transportation, Wuhan is known as the “thoroughfare to nine provinces” in name and in fact and has been awarded the name “the Chicago of China” [40]. Wuhan consists of 17 administrative districts, including 7 central urban districts, 6 new urban districts and 4 development zones. Moreover, 3493 URAUs are divided up based on the current urban and rural grassroots management system, and these units include 1392 urban communities and 2101 villages. Figure 1 shows the elderly population density of the URAUs in Wuhan city in 2016.

### 2.2. Study Data and Preprocessing

**Figure 2:**
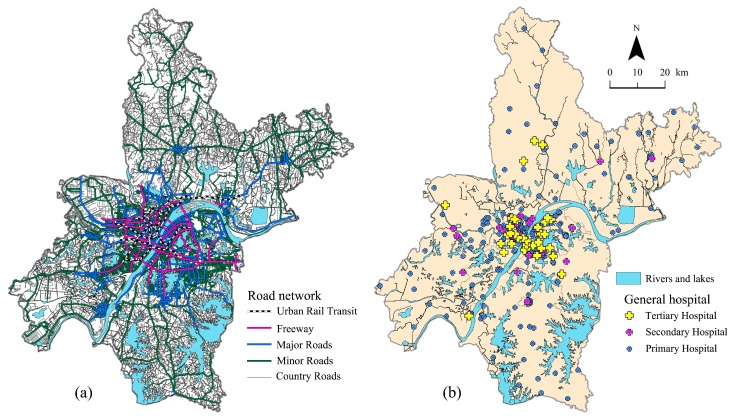
Road network (**a**) and location of selected general hospitals (**b**) in Wuhan city.

The study data were derived from the first National Geographic Conditions Monitoring (NGCM) of Wuhan executed in 2016 and included three categories: ageing population data, road network data and general hospital data. Data sources and preprocessing instructions were as follows:

1. Ageing population data. A GIS format vector polygon layer extracted from the NGCM includes 3493 URAUs, and each URAU has age-specific demographic data. As shown in Figure 2, a population map of Wuhan was drawn covering all URAUs.
2. Road network data included urban rail transit, freeways, major roads, minor roads and country roads. As shown in Figure 2a, a vector road network data of Wuhan was built up after topology checking. The average speed set for different road types refers to the Code for Design of Urban Road Eengineering (CJJ37-2012), which is one of the national standards of China. Table 1 shows the speed stability and characteristics of different kinds of roads in Wuhan, China.
3. General hospital data included vector GIS format and volume data. The vector data are the hospitals registered in the health department of Wuhan, and the point vector data were obtained by vectorization according to the registered name and address. Data preprocessing of the general hospital primarily consisted of two aspects: selecting medical facilities and measuring the medical services supply volume. For the elderly, with increasing age, numerous underlying physiological changes occur, and the risk of disease rises [41]. Considering that common sudden diseases (e.g., ischaemic heart disease, stroke, hypertensive heart disease, etc.) and severe injuries of the elderly population are comprehensive and emergent, clinics and specialized hospitals, such as rehabilitation hospitals, children’s hospitals, stomatological hospitals, and cosmetic surgery hospitals, cannot satisfy the elderly medical service demand entirely. Hence, general hospitals, which are set up to address many types of disease and injuries and normally have an emergency department were selected in our study after eliminating clinics and specialized hospitals. General hospitals in China can be roughly divided into three types: primary hospitals, secondary hospitals and tertiary hospitals [42]. In 2016, there were 365 hospitals in Wuhan city, and as is shown in Figure 2b, 288 general hospitals have been selected based on the above filters, including 213 primary hospitals, 40 secondary hospitals and 35 tertiary hospitals.

The number of hospital beds and professional physicians are the two main measurement indicators of hospital medical services according to China’s current standards. To a certain extent, beds represent the hardware facility scale of medical institutions, and professional physicians can reflect the quality of medical services. Hence, the medical services comprehensive supply volume (MSCSV) of hospitals were measured by the number of hospital beds and professional physicians in this study (Table 2). The two indicator statistics were mainly derived from the registration information in the National Health Commission (NHC) of China, partly from the official website of each hospitals.

To calculate the hospital’s MSCSV, the weighted average method was used to pretreat the two indicators. Zhang presented the analytic hierarchy model (AHP) to measure the ability of public hospital medical services, and the results showed that the indicator weights of “professional physicians” and “hospital beds” were 0.258 and 0.171, respectively [44]. Accordingly, the weights of the number of hospital beds and professional physicians were 0.33 and 0.67, respectively, and the MSCSV of each hospital was calculated.

## 3. Methodology

**Figure 3:**
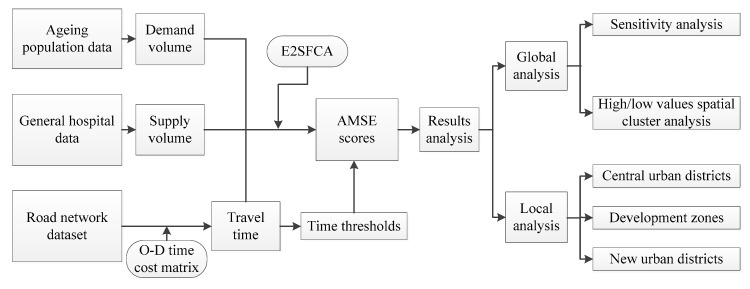
Research process flow chart; E2SFCA: the road network-based Guassian 2SFCA method; O–D: origin to destination.

First, the medical service’s demand and supply volume were extracted from the ageing population data and general hospital data, respectively. Second, assuming that the automobile (i.e., driving or taking a car) and urban rail transit were the two optional means of transportation for elderly people in Wuhan to obtain medical services, the travel time was calculated by the O–D time cost matrix based on the road network analysis in ArcMap 10.3 (ESRI, 380 New York Street, Redlands, CA, USA). Next, the AMSE scores were calculated using E2SFCA after the catchment area was defined. Last, global and local analyses were conducted to comprehensively illustrate the spatial differences of AMSE scores in the results analysis part. The global analysis included two dimensions: sensitivity and high/low value spatial cluster analysis. In addition, the AMSE scores of three regional areas were used as an internal comparison in local analysis. The process flow of our research is shown in Figure 3.

### 3.1. Enhanced Two-Step Floating Catchment Area (E2SFCA) Algorithm

A road network dataset was created based on different road types, as shown in Table 1. Assuming that the automobile (e.g., driving or taking a car) and urban rail transit are the prior selected transportation vehicles for the elderly to get to hospitals, the travel times from URAUs (i.e., Origin) to general hospitals (i.e., Destination) were calculated using the O–D time cost matrix. Based on the travel times, the E2SFCA method adding a Gaussian function to the model time decay effect was used. The attenuation in E2SFCA presents roughly an *S* shape; that is, with time increasing, the attenuation speed rate of the accessibility increases from slow to fast, and then slows down again. A Gaussian function *G*(*t_kj_*,*t*_0_) is expressed as follows:

(1) $$G(t_{kj},t_{0})=\left\{ \begin{array}{l} \frac{e^{-(\frac{1}{2})\times {\left(\frac{t_{kj}}{t_{0}}\right)}^{2}}-e^{-(\frac{1}{2})}}{1-e^{-(\frac{1}{2})}},t_{kj}\leq t_{0}\\ 0,t_{kj}>t_{0} \end{array}\right.$$

where *t_kj_* is the time cost between the general hospital location *j* (i.e., medical service supply) and URAU locations *k* (i.e., medical service demand), and *t*_0_ is a certain threshold travel time.

Step 1: define the catchment of location *j* as an area composed of all locations *k* within a threshold travel time *t*_0_ from *j*. The medical services supply-demand ratio (*R_j_*) within the catchment area was computed as:

(2) $$R_{j}=\frac{S_{j}}{\sum_{k\in \left\{t_{kj}\leq t_{0}\right\}}G(t_{kj},t_{0})D_{k}}$$

where *S_j_* is the measure of MSCSV at location *j*; *G*(*t_kj_*,*t*_0_) is the time weight for the threshold travel time zone calculated from the Gaussian function, capturing the time decay of access to the general hospital *j*, and 0 < *t_kj_*≤ *t*_0_. *D_k_* is the ageing population medical services demand volume at location *k*.

Step 2: For each URAU location *k*, all hospitals locations (*j*) that were within a certain threshold travel time *t*_0_ zone from location *k* were searched, and the medical services supply–demand ratios *R_j_* (calculated in step 1) were summed. Meanwhile, the same distance weight derived from the Gaussian function used in Step 1 was applied to a certain threshold travel time *t*_0_ zone for time decay as follows:

(3) $$AMSE_{k}=\sum_{j\in \left\{t_{kj}\leq t_{0}\right\}}G(t_{kj},t_{0})R_{j}$$

where *AMSE_k_* represents the final accessibility score assigned to the URAU at location *k*.

### 3.2. Two-Threshold Travel Time

To differentiate accessibility within a catchment, two threshold travel time zones within each catchment were obtained using the ArcGIS Network Analyst. The “Platinum Ten” is a concept familiar to all types of emergency rescue and ambulance crews and is the period during which emergency crews, upon their arrival at the scene, assess the situation and initiate treatment and transport of casualties. The “Golden Hour” has been employed in rescue for more than two decades. It is the hour that you have to get someone to advanced medical treatment in order to increase their chances of survival [45,46]. In this paper, two important emergency first aid times (i.e., 10 min and 60 min) were used as the threshold travel time.

### 3.3. Hot Spot Analysis (Getis-Ord G_i_*)

To assess whether and where high or low AMSE scores cluster spatially, the hot spot analysis tool in ArcMap 10.3 was used to calculate the Getis-Ord *G*_i_^*^ statistic for each URAU in a dataset. The resultant *Z*-scores and *p*-values indicated where URAUs with either high or low values (i.e., AMSE scores) clustered spatially. A high *Z*-score and small *P*-value for a URAU indicated a significant hot spot. A low negative *Z*-score and small *P*-value indicated a significant cold spot. The higher (or lower) the *Z*-score, the more intense the clustering. A *Z*-score near zero means no spatial clustering. The Getis–Ord local statistic is given as:

(4) $$G_{i}^{*}=\frac{\sum_{i=1}^{n}w_{i,j}x_{j}-\bar{X}\sum_{j=1}^{n}w_{i,j}}{S\sqrt{\frac{\left[n\sum_{j=1}^{n}w_{i,j}^{2}-{\left(\sum_{j=1}^{n}w_{i,j}\right)}^{2}\right]}{n-1}}}$$

where *x_j_* is the attribute value for URAU *j*; *w_i_*_,*j*_ is the spatial weight between URAU *i* and *j*; *n* is the number of URAUs in the dataset and URAUs *i* and *j* cannot be the same URAU. The *Z*-score for the statistic was computed as:

(5) $$S=\sqrt{\frac{\sum_{j=1}^{n}x_{j}^{2}}{n}-{\left(\bar{X}\right)}^{2}};\;\bar{X}=\frac{\sum_{j=1}^{n}x_{j}}{n}$$

The *G*_i_^*^ statistic is a *Z*-score so no further calculations were required.

## 4. Experimental Results

### 4.1. Global Analysis of Accessibility of Medical Services for the Elderly (AMSE) Scores

#### 4.1.1. Sensitivity Analysis

**Figure 4:**
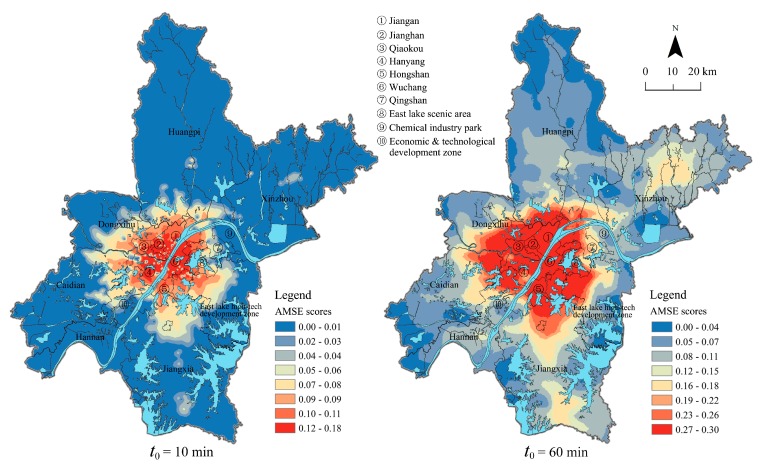
Spatial AMSE scores for two different threshold travel times.

**Figure 5:**
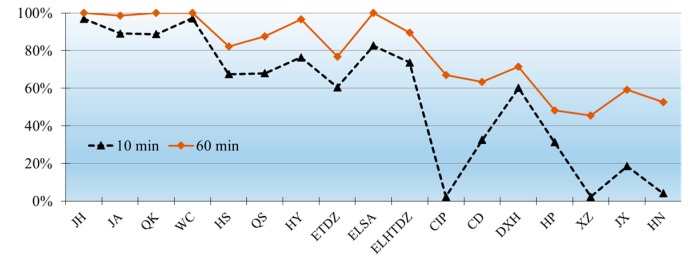
The proportion of the elderly population at higher- or the higest level medical accessibility in each district; capital letters in abscissa denote the abbreviations of different district in Figure 1.

As is shown in Figure 4, the AMSE scores in two different threshold travel times referred to in Section 3.1 were calculated using Equation (3). According to the distribution of AMSE scores under the two different time thresholds, Jenks Natural Breaks Classification was used to divide the scores into five categories (e.g., the lowest-, lower-, medium-, higher- and the highest-level) in Table 3. Meanwhile, the proportion of the elderly population at higher- or the highest level medical accessibility in each district is shown in Figure 5.

When the time threshold *t*_0_ = 10 min, AMSE scores in Wuhan presented a “cluster” spatial distribution pattern, which was mainly affected by the urban road network. The statistics shows that there were 955 URAUs with 84.01 million elderly people who had higher-or the highest level medical accessibility, and these elderly people were mainly distributed in the central urban area. The vast majority of older adults in Jianghan and Wuchang district had a higher-or the highest level accessibility to pubulic general hospitals. However, very small proportions of the older adults in Huangpi, Xinzhou, Hannan, Jiangxia, Chemical industry park and Caidian district had considerable access to healthcare in 10 minutes, which means that more medical resources should be distributed in these areas as soon as possible and more specialized Fast Tracks should be built for the elderly‘s urgent care.

When the time threshold was *t*_0_ = 60 min, AMSE scores obviously increased, which was highly consistent with the spatial distribution of the external major roads. In addition, there were 1566 URAUs with more than 128.31 million elderly people that had higher- or the highest level medical accessibility. The place had more developed external major roads when the AMSE scores were higher. According to the statistics, most of the elderly people lived in the central urban areas had higher or the highest level medical accessibility, but nearly half of the older adults in HP and XZ district had little access to healthcare in 60 min because of a lack of medical resources and poor traffic conditions.

#### 4.1.2. High/Low Value Spatial Cluster Analysis

**Figure 6:**
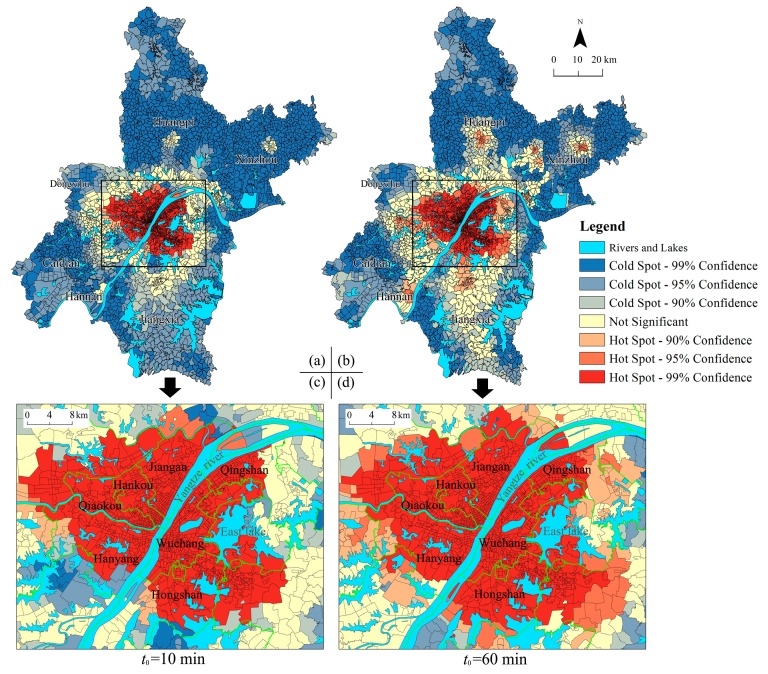
Hot spot Analysis of urban-rural resident autonomous units (URAUs) for Wuhan; (**a**,**b**) is the map in the global perspective as threshold time *t*_0_ = 10 min and *t*_0_ = 60 min correspondingly; (**c**,**d**) is the central urban area in an enlarged view with threshold time *t*_0_ = 10 min and *t*_0_ = 60 min correspondingly.

The Getis–Ord *G*_i_^*^ statistics of two different threshold travel times were calculated using Equation (4), and then they were divided into three categories; that is, hot spot, not significant area and cold spot, based on the Jenks Natural Breaks method. The hot to cold rendering in the two threshold travel times are shown in Figure 6. The results showed that hot spots were located in the central urban areas, and the spatial pattern of AMSE scores in Wuhan city presented an enclosure structure in general. In other words, the URAUs in the city centre area had high-high values cluster spatially because of its significant positive *Z*-scores and larger *Z*-score and presented a “hot spot–not significant area–cold spot” annular distribution pattern from the city centre area to the periphery correspondingly. According to the statistics, 1086 URAUs were located in the hot spots, 309 URAUs were not significant and 2098 URAUs were located in the cold spots at *t*_0_ = 10 min. In addition, the Jiangan, Hankou, Qiaokou, Wuchang districts and most parts of Hanyang, Hongshan, and Qingshan districts were located in the hot spots. However, most parts of new urban districts were located in the cold spots.

When *t*_0_ = 60 min, the hots pots of AMSE scores were clustered in the central urban areas, and several secondary hot spots were distributed in the built-up area of the new urban area. Meanwhile, 1438 URAUs were located in the hot spots, 430 URAUs are not significant, and 1625 URAUs were located in the cold spots. Qingshan district is a new hot spot area, and most parts of Hanyang and Hongshan were located in the hot spots.

### 4.2. Local Analysis of AMSE Scores

#### 4.2.1. Central Urban Districts

**Figure 7:**
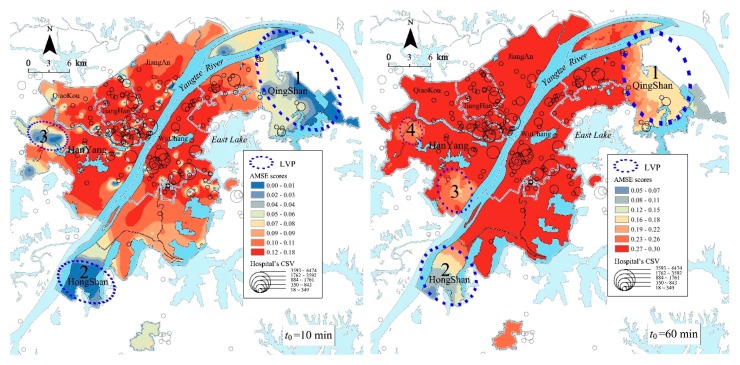
Spatial accessibility of medical services for the elderly (AMSE) scores, general hospital’s medical services comprehensive supply volume (MSCSV) and three low-value plaques (LVPs) of central urban districts for the two threshold times.

**Figure 8:**
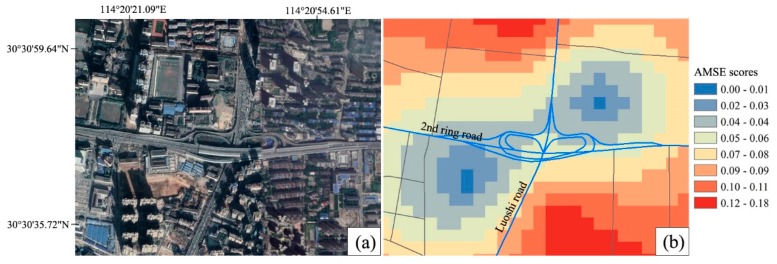
(**b**) A small LVP of AMSE scores occurred near an overpass located at the intersection 2nd ring road and Luoshi road in Hongshan district as *t*_0_ = 10 min; the image in (**a**) was extracted from Google Earth.

Because of the highest road density and the largest hospital’s MSCSV, the elderly in the central urban district had the highest AMSE scores. As is shown in Figure 7, when *t*_0_ = 10 min, the areas with higher or the highest level (i.e., the scores were 0.08–0.18) AMSE scores are mainly distributed in Jianghan, Wuchang and Hanyang districts. However, three major low-value plaques (LVPs) existed in the marginal area of central urban districts: the biggest was located in the north-east of the central urban districts, which were mostly in Qingshan district, the second was distributed in the south of Hongshan district and the third was located in Hangyang district in the west of the central urban districts. Meanwhile, because of the barrier effect of controlled-access highways, there were also many small LVPs in central urban districts. The effect makes it difficult for elderly people living near overpasses and ramps to get medical services quickly as the controlled-access highways have no traffic signals, intersections or property access. Figure 8 shows a small LVP of AMSE scores thtoccurred near an overpass located at the intersection 2nd ring road and Luoshi road in Hongshan district.

When *t*_0_ = 60 min, the AMSE scores in the majority area of central urban districts were generally at a higher- or the highest level (i.e., the scores were 0.12–0.30), and the internal differences were small. However, there were four LVPs in the marginal area. Similarly, the biggest plaque was mostly distributed in Qingshan district and the second was located in the south of Hongshan district. The difference was that Hangyang district has two LVPs, in which the AMSE scores were at a higher level.

#### 4.2.2. Development Zones

Development zones have lower AMSE scores compared to central urban districts. Although elderly people that lived near the central urban area had greater medical accessibility, some remote URAUs were blocked by natural geographical factors, such as rivers and large lakes, which led to spending more travel time to obtain medical services. For instance, the elderly people who lived in Chemical industry park had to spend more time in traffic due to the natural barriers, not only because of the Yangtze River in the north but also the East Lake in the south.

**Figure 9:**
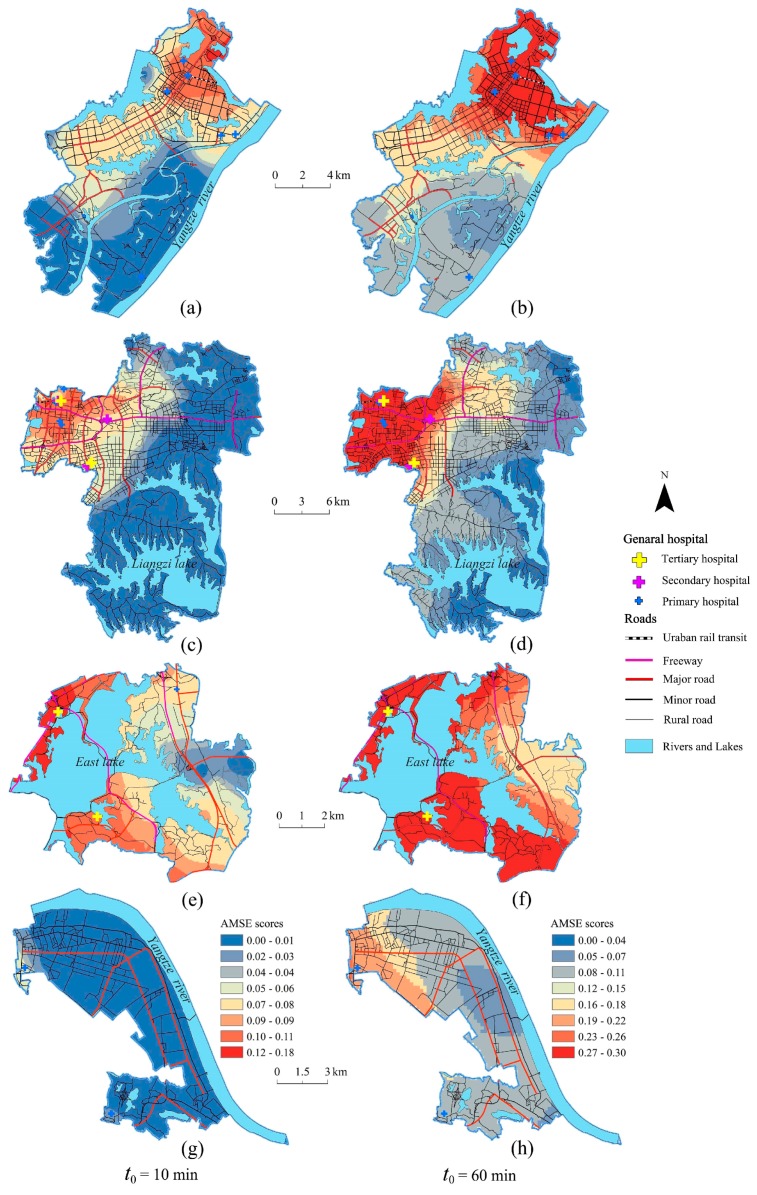
Spatial distribution of AMSE scores in different development zones. (**a**,**b**) are the images of AMSE scores in Economic & technological development zone (ETDZ); (**c**,**d**) are the images of AMSE scores in East lake high-tech development zone (ELHTDZ); (**e**,**f**) are the images of AMSE scores in East lake scenic area (ELSA); (**g**,**h**) are the images of AMSE scores in Chemical industry park (CIP).

The spatial differences of AMSE scores in development zones were obvious. Specifically, the AMSE scores in the north ETDZ were significantly higher than that of the south. The reason is that the roads in the north are more developed than in the south, and the north has a higher road density. In addition, several primary hospitals are concentrated in the north, while only one primary hospital is located in the south. As shown in Figure 9a,b, the AMSE scores in ETDZ are generally at a lower or lowest level, except for a small part of the north, which had a medium level as *t*_0_ = 10 min, and the AMSE scores were generally at a medium-and above level at *t*_0_ = 60 min. The AMSE scores in north-west East lake high-tech development zone ELHTDZ were significantly higher than in other areas because general hospitals are mainly distributed in the northwest, the road network is dense and the traffic is convenient. As is shown in Figure 9c,d, the AMSE scores in ELHTDZ were generally at a lowest level at *t*_0_ = 10 min; specifically, the elderly people lived in the URAUs far from the general hospitals, where it is difficult to get medical services within 10 min. Meanwhile, the AMSE scores in ELHTDZ were generally at a medium- and-above level at *t*_0_ = 60 min; however, fringe areas in the east, such as those on the east coast of Liangzi lake, are places where it is still difficult to conveniently obtain medical services. Compared to other development zones, East lake scenic area ELSA generally has the highest level of AMSE scores. This finding is observed primarily because ELSA is close to the central urban area where medical services are convenient to access, and two tertiary hospitals can provide a large number of medical services. As is shown in Figure 9e,f, the AMSE scores in ELSA were generally at a medium- and-above level at *t*_0_ = 10 min, and at a higher- or the highest level at *t*_0_ = 60 min. The level of AMSE scores in CIP is generally low. It is difficult for the elderly to obtain medical services effectively, mainly because CIP is far away from the central urban area and contains few general hospitals. As shown in Figure 9g,h, the AMSE scores of CIP were generally at a lower- or medium level at *t*_0_ = 60 min, and showed an obvious increase compared to that of *t*_0_ = 10 min.

#### 4.2.3. New Urban Districts

Comparatively speaking, new urban districts had the lowest AMSE scores, and only the elderly in some urban areas enjoyed better medical services. The rural areas in the new urban districts were distant from the urban areas, and the traffic was inconvenient, which may cause the elderly in the new urban districts to face a shortage of medical resources. 

**Figure 10:**
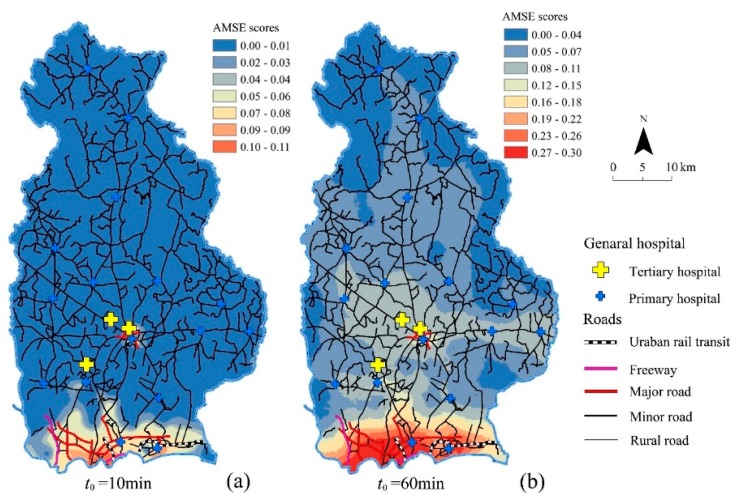
AMSE scores and topographic map of Huangpi district. (**a**) shows the AMSE scores at *t*_0_ = 10 min; (**b**) shows the AMSE scores at *t*_0_ = 60 min.

Taking Huangpi district as an example, Huangpi is located in the joint part of the Dabie mountain area, Jianghan plain and hilly region in north-east Hubei province. As shown in Figure 10a, Huangpi had the lowest level AMSE scores in most areas when the threshold travel time *t*_0_ was 10 min, and only a small area near the central urban district had lower- or medium-level AMSE scores. As is shown in Figure 10b, Huangpi had lower- or medium-level AMSE scores in most areas at *t*_0_ = 60 min, and the place in southern Huangpi had higher- or the highest level AMSE scores. The Huangpi district had the largest number of elderly people, however, it contained few general hospitals. In addition, the topography of Huangpi is varied and complicated because many low hills are located in northern Huangpi, and the altitude varies from 17 to 873 m.

## 5. Discussion

### 5.1. The Innovation of Measuring the AMSE Method

Spatial access emphasizes the importance of spatial separation between supply (i.e., healthcare providers) and demand (i.e., population) and how they are connected in space [47] and thus is a classic issue for location analysis well suited for GIS to address. For many years, multiple methods have been used to measure the spatial accessibility of medical services, for example, the supply–demand match ratio [48], the gravity-based accessibility model [49] and the two-step floating catchment area (2SFCA) method [21,22,23,24,25,26,27]. The first one is a simple method which neither reveals the detailed spatial variations within an area unit (e.g., a county or a subcounty area) nor accounts for interaction between population and physicians across areas. The latter two methods considered the factors of traffic time and distance from demand side to supply side as well as the scale of both sides. Compared to the gravity model, the 2SFCA method is more intuitive to interpret and easier to calculate [18,19,20].

The original 2SFCA method has drawn sharp criticism because of its dichotomous approach that defines a doctor inside a catchment as accessible and one outside the catchment as inaccessible [25]. Several studies have attempted to improve the 2SFCA method; for example, a kernel density function [21] or a Gaussian function [29,30] have been proposed to model the distance decay effect. Dai et al. (2010) indicated that the Gaussian 2SFCA method performs better than the conventional 2SFCA. Meanwhile, considering that older adults are typically less likely to travel long distances [31], specifically, they are more likely to obtain medical care near their residence. This phenomenon can be expressed mathematically as a Gaussian function in distance (or time) decay mechanism.

To the best of our knowledge, notably little research has been conducted to measure the accessibility of the ageing population-focused medical services using the Gaussian 2SFCA method, and in particular, very few studies of AMSE have considered the road network with different road types when calculating the travel times. Therefore, our study proposed a road-network-based Gaussian 2SFCA (E2SFCA) method for measuring the AMSE.

### 5.2. The Division of Travel Time Zones

The catchment radius of E2SFCA might also vary by provider types or neighborhood types [23]. Weights can be assigned to different travel time zones to account for the distance decay effects within each catchment area [50]. Considering that common sudden diseases and severe injuries of the elderly population are comprehensive and emergent, some studies have defined the two threshold travel times (i.e., Platinum Ten and Golden Hour) in relevent practical studies [46,51].

The elderly, as a vulnerable population, are more likely to have sudden diseases and severe injuries than young adults. Hence, it would be of greater significance for attaching the two important emergency first aid times to calculate the accessibility of medical services for the elderly.

### 5.3. Traffic Conditions Affect Car Travel Time

In this study, the AMSE scores were calculated using several static average speeds, Table 1 shows the different road types were divided into two categories: stable and unstable speed roads. However, traffic conditions in 24 h or holiday seasons are quite different, namely the changeable car travel time will lead to the instability of the AMSE scores. For example, the AMSE scores in some part of central urban areas may decrease in rush hours (e.g., 7:15 a.m.–8:45 a.m. and 17:05 p.m.–19:00 p.m.) and then it will increase in normal hours. Despite ambulances having priority access through intersections, the time would increase because some roads usually have traffic congestion in rush hours and car accidents may occur occasionally. Therefore, in future studies, dynamic analysis of AMSE scores is also needed by dividing the time into several categories.

## 6. Conclusions

As the proportion of elderly people increases worldwide, assessing geographical accessibility to general hospitals become particularly important. This article aimed to measure AMSE using an enhanced 2SFCA method. Based on the travel time for elderly people to get medical services, the E2SFCA was used to measure AMSE in Wuhan, China. Next, in order to comprehensively illustrate the characteristics of AMSE scores, the analysis results were divided into two parts:

In terms of global analysis, two conclusions can be drawn: Firstly, sensitivity results showed that 48.63% of the elderly population in Wuhan had higher or the highest level medical accessibility at *t*_0_ = 10 min, and 72.97% had higher or the highest level medical accessibility at *t*_0_ = 60 min. Secondly, high/low value spatial cluster analysis showed that Jiangan, Hankou, Qiaokou, Wuchang districts and most parts of Hanyang, Hongshan, and Qingshan districts were located in the hot spots at the two threshold times, and indicates that these districts were the main areas providing medical services for the elderly. However, compared to other districts, most parts of new urban districts were located in the cold spots, which indicates that more medical resources should be distributed in these areas as soon as possible and more specialized fast tracks should be built for the elderly‘s urgent care.

The local analysis led to the following conclusions: (1) the AMSE scores in the three kinds of administrative districts basically satisfied the relationship: central urban districts > development zones > new urban districts; (2) several major low-value plaques (LVPs) existed in the marginal area of central urban districts. Particularly, many small LVPs occurred in central urban districts at *t*_0_ = 10 min because of the barrier effect of controlled-access highways; and (3) several natural barriers (i.e., low hills, rivers and lakes) in development zones and new urban districts could lower the surrounding AMSE scores. Meanwhile, multiple approaches, such as improving traffic conditions, optimizing the general hospital’s location, appointing more professional physicians or adding more hospital beds can increase accessibility.

Overall, the study on measuring and analysing AMSE may help to understand the seriousness of the ageing problem from the perspective of medical care. The accessibility results identifying the hot spots for elderly medical services provided spatial references for medical services’ optimal allocations. To simulate the time of health emergencies that occur in the elderly, the results of AMSE scores calculated at the two threshold travel times were analysed. It is possible that the E2SFCA method is also worth promoting when measuring accessibility to other facilities (e.g., banks, libraries, churches, and shopping centres) for elderly people. Additionally, our work may facilitate further investigation into the accessibility of medical services for other vulnerable populations (e.g., women, children, and the disabled) in China and other countries.

## Figures and Tables

**Table 1:** Speed stability and characteristics of different road types in Wuhan, China.

Road Types		Average Speed (km/h)	Speed Stability	Descriptions	Characteristics
Urban roads	Urban rail transit (URT)	40	Stable	Metro; light rail transit (LRT)	No traffic jams and traffic lights
	Freeway	60		Urban high-speed road; ring road	Seldom have traffic jams; no traffic lights
	Major roads	55	Unstable	Arterial road; secondary road	Sometimes have traffic jams; many traffic lights
	Minor roads	40		Tertiary road; fourth-class road; branch road	Sometimes have traffic jams; fewer traffic lights
Rural roads		30	Stable	Rural hardened road	Seldom have traffic jams; no traffic lights

**Table 2:** Major characteristics of general hospitals in current China [43].

General Hospitals	Scales		Levels	Medical Services
	Hospital Beds	Professional Physicians (Per Bed)		
Primary hospital	20–99	0.70	Community-level	Providing prevention, treatment, healthcare and rehabilitation services.
Secondary hospital	100–499	0.88	County-level	Providing comprehensive medical and health services.
Tertiary hospital	≥500	1.03	Region-level or nationwide	Providing high-level specialized medical and health services.

**Table 3:** Statistics and classification of accessibility of medical services for the elderly (AMSE) scores at two time thresholds.

Time Threshold *t*_0_	Level	AMSE Scores	Number of URAUs	Proportion of URAUs	Elderly Population (Ten Thousands)	Proportion of Elderly Population
10 min	The lowest	0.00–0.01	1639	46.92%	46.89	27.14%
	Lower	0.02–0.04	376	10.76%	17.21	9.96%
	Medium	0.05–0.07	523	14.97%	24.63	14.26%
	Higher	0.08–0.11	483	13.83%	39.82	23.05%
	The highest	0.12–0.18	472	13.51%	44.19	25.58%
60 min	The lowest	0.00–0.04	412	11.80%	9.07	5.16%
	Lower	0.05–0.08	891	25.51%	21.51	12.23%
	Medium	0.09–0.11	624	18.44%	16.96	9.64%
	Higher	0.12–0.18	557	15.37%	29.87	16.99%
	The highest	0.19–0.30	1009	28.89%	98.44	55.98%
